# Comparison of preoxygenation efficiency measured by the oxygen reserve index between high-flow nasal oxygenation and facemask ventilation: a randomised controlled trial

**DOI:** 10.1186/s12871-023-02126-9

**Published:** 2023-05-09

**Authors:** Sujung Park, So Yeon Kim, Min-Soo Kim, Wyun Kon Park, Hyo-Jin Byon, Hyun Joo Kim

**Affiliations:** Department of Anesthesiology and Pain Medicine, Anesthesia and Pain Research Institute, Yonsei University College of Medicine, Seoul, South Korea

**Keywords:** Anesthetic induction, Apneic oxygenation, Facemask ventilation, High-flow nasal cannula, High-flow nasal oxygenation, Oxygen reserve index, Preoxygenation

## Abstract

**Background:**

High-flow nasal oxygenation and the oxygen reserve index (ORI), which is a non-invasive and innovative modality that reflects the arterial oxygen content, are used in general anaesthesia. This study compares the preoxygenation efficiency (measured by the ORI) of high-flow nasal oxygenation and facemask ventilation during the induction process.

**Methods:**

This single-centre, two-group, randomised controlled trial included 197 patients aged ≥ 20 years who underwent orotracheal intubation for general anaesthesia for elective surgery. The patients were randomly allocated to receive preoxygenation via facemask ventilation or high-flow nasal oxygenation. The ORI was measured and compared between both groups.

**Results:**

The ORI increased during preoxygenation in all patients. At 1 min of preoxygenation, the ORI was significantly higher in the high-flow nasal oxygenation group (0.34 ± 0.33) than in the facemask ventilation group (0.21 ± 0.28; P = 0.003). The highest ORI was not significantly different between the two groups (0.68 ± 0.25 in the high-flow nasal oxygenation group vs. 0.70 ± 0.28 in the facemask ventilation group; P = 0.505).

**Conclusions:**

High-flow nasal oxygenation results in an oxygenation status similar to that provided by facemask ventilation during the induction process of general anaesthesia; therefore, high-flow nasal oxygenation is a feasible preoxygenation method.

**Trial Registration:**

Clinicaltrials.gov (NCT04291339).

## Background

Endotracheal intubation requires an anaesthetic induction process that results in loss of consciousness and neuromuscular block. This process includes hypoventilation and apnoea, resulting in a risk of hypoxia, which further exacerbates in case of difficult endotracheal intubation [1]. Therefore, careful preoxygenation before anaesthesia induction is recommended when a patient is breathing spontaneously [2].

The conventional preoxygenation method includes the use of a facemask, which allows patients to spontaneously breathe oxygen [2]. The Optiflow device (Fisher & Paykel Healthcare, Auckland, New Zealand) supplies heated and humidified oxygen through a nasal cannula. This high-flow nasal oxygenation (HFNO) method allows apnoeic oxygenation and improves atelectasis. It has gained popularity owing to its ease of use and potential for hands-free anaesthesia induction [3]. It also increases the end-expiratory lung volume and improves dynamic compliance [4].

In previous studies, HFNO in patients undergoing anaesthesia induction for emergency surgery resulted in similar arterial oxygen partial pressure (PaO_2_), oxygen saturation (SpO_2_), and desaturation incidence compared to standard facemask oxygenation immediately after endotracheal intubation, suggesting that HFNO is an effective preoxygenation method [5–7]. However, these studies were either conducted with rapid sequence induction, without mask ventilation being performed during the facemask oxygenation, or with endotracheal intubation being conducted 1–2 min after the administration of hypnotics and neuromuscular blocking drugs. Therefore, these data do not reflect the general anaesthetic induction process of patients undergoing non-emergent surgery.

A previous study on non-rapid sequence induction demonstrated that the post-endotracheal intubation PaO_2_ was significantly lower with HFNO than with facemask oxygenation [8]. Another study reported no significant difference in the incidence of SpO_2_ < 92% during anaesthesia induction [9]. Therefore, the efficacy of HFNO for non-rapid sequence induction is controversial. Moreover, prior studies did not implement manoeuvres such as jaw thrust, which are recommended for maintaining upper airway patency with HFNO.

The oxygen reserve index (ORI™, Masimo Corp., Irvine, CA) continuously monitors the amount of oxygen non-invasively, similar to what is done for SpO_2_. The ORI reflects a PaO_2_ range of 80–200 mmHg as a value between 0 and 1 [10]. The ORI enables the control of preoxygenation and provides an early warning of hyperoxia or desaturation without monitoring end-tidal oxygen concentration through a ventilator device [11]. Therefore, the ORI was used in this study to investigate the preoxygenation efficiency of HFNO and facemask oxygenation during anaesthesia induction.

In this randomised controlled study, we hypothesized that HFNO would have higher preoxygenation efficiency than facemask oxygenation in adult patients undergoing general anaesthetic induction for non-emergent surgery. The ORI was monitored during the anaesthesia induction period, and the highest ORI values obtained using HFNO and facemask oxygenation were compared.

## Methods

### Patients

This prospective, single-centre, randomised controlled trial was performed at Severance Hospital, Yonsei University Health System, Seoul, South Korea. This study was approved by the institutional review board of Severance Hospital, Yonsei University Health System (50 − 1 Yonsei-ro, Seodaemun-gu, Seoul, Korea; # 4-2019-1336) and written informed consent was obtained from all individuals participating in the trial. The trial was registered prior to patient enrolment at clinicaltrials.gov (NCT04291339, Principal investigator: Hyun Joo Kim, Date of registration: 02/03/2020). The study protocol followed applicable SPIRIT (Standard Protocol Items: Recommendations for Interventional Trials) guidelines. This study was conducted in accordance with the Declaration of Helsinki and the relevant guidelines.

### Inclusion and exclusion criteria

Patients aged ≥ 20 years who were scheduled to undergo orotracheal intubation for general anaesthesia from November 2020 to January 2021 were enrolled in this study. Patients with cranial fractures or craniofacial malformations that prevented oxygen delivery through the nostrils, those who could not cooperate due to impaired consciousness, and those who could not fast prior to surgery or were at risk of aspiration of gastric contents due to gastroesophageal disease were excluded from the study. Patients who required awake intubation were also excluded.

### Randomisation

A computer-generated randomisation table (available at https://www.randomizer.org/form.htm) was used to randomly assign patients to the HFNO group or the facemask ventilation (FMV) group at a 1:1 ratio. Due to differences in the appearance of the preoxygenation devices, the patients and anaesthesiologists could not be blinded to group allocation once the induction process was initiated. Prior to surgery, group allocations were concealed in sequentially numbered, sealed, opaque envelopes. Randomisation was performed after enrolment but before the start of the study protocol by Hyun Joo Kim.

### Anaesthetic management and measurements

In the operating room, patients were placed in a supine position with a pillow behind their head. An electrocardiogram was obtained, and blood pressure, oxygen saturation, and brain function (SedLine®, Masimo Corp., Irvine, CA, USA) were monitored non-invasively. Each patient’s pulse oximetry was measured using the RD Rainbow SET®-2 Neo sensor (Massimo Corp.) attached to the patient’s finger and covered with a light-shielding black bag. The ORI was monitored using the Rainbow SET Radial-7 Pulse CO-Oximeter (software version 2.1.3.5; Masimo Corp.).

Prior to initiating anaesthesia induction, an airway assessment was performed, including assessments for obstructive sleep apnoea, snoring while sleeping, interincisor distance, hyomental distance, and head and neck movement. The modified Mallampati and upper lip bite test scores were also determined.

In the HFNO group, the Optiflow (Fisher & Paykel Healthcare, Auckland, New Zealand) nasal cannula was placed on the patient’s nares. Cannula size was adjusted with respect to the nasal opening. Preoxygenation was performed for 3 min at 40 L·min^− 1^ at an FiO_2_ of 100%. The temperature of Optiflow was set to 37℃ for all patients. During the preoxygenation period, the patient was asked to rate whether they were comfortable with the oxygen administration method using a four-level scale (comfortable, acceptable, uncomfortable, and intolerable). If the patient complained of nasal discomfort, the oxygen flow rate was reduced in increments of 5 L·min^− 1^ to a minimum of 30 L·min^− 1^.

After preoxygenation, anaesthesia induction was accomplished intravenously using propofol (1–2 mg·kg^− 1^). Intravenous rocuronium (0.6 mg·kg^− 1^) is used after the patient lost consciousness in both groups. Oxygen was administered via nasal cannula at 70 L·min^− 1^ for 3 min, and the patient’s jaw was lifted using the operator’s hands to maintain upper airway patency. After rocuronium administration, endotracheal intubation was attempted using a video laryngoscope and an endotracheal tube. The high-flow nasal cannula was used to continuously supply oxygen at 70 L·min^− 1^ until the endotracheal tube was placed.

In the FMV group, the facemask was placed in contact with the patient’s nose and mouth, and oxygen was supplied at 10 L·min^− 1^ at an FiO_2_ of 1.0 for 3 min. During preoxygenation, the patient’s comfort level was assessed using the same scale as that used in the HFNO group. If discomfort was reported, the oxygen flow rate was lowered in increments of 1 L·min^− 1^ to a minimum flow rate of 6 L·min^− 1^. When the patient lost consciousness, facemask oxygenation was continued for 3 min using the volume-controlled mode of the mechanical ventilator with an oxygen flow rate of 2 L·min^− 1^, tidal volume of 8 mL·kg^− 1^, respiratory rate of 15 breaths per minute, peak end-expiratory pressure of 0 cmH_2_O, and FiO_2_ of 1.0. A two-handed jaw thrust manoeuvre was performed to maintain upper airway patency. Endotracheal intubation was performed using a video laryngoscope 3 min after the administration of rocuronium. The mask was removed from the patient’s face, and oxygenation was discontinued during intubation.

The ORI was continuously monitored until the endotracheal intubation was completed. ORI and SpO_2_ values were collected at 10 time points: at baseline (before preoxygenation); at 1, 2, and 3 min of preoxygenation; during the propofol and rocuronium injections; at 1 and 2 min after the rocuronium injection; and at the initiation and completion of intubation.

Patient characteristics such as age, sex, height, weight, and the American Society of Anesthesiologists physical class were recorded. The number of intubation attempts, intubation time, and Cormack–Lehane grade were recorded. The primary outcome was the highest ORI value achieved by oxygenation. The secondary outcome was the time required to reach the highest ORI observed during the induction of anaesthesia.

### Sample size calculation

Data from previous studies were used to calculate the sample size [12]. The highest mean ORI value during anaesthesia induction was estimated as 0.5 ± 0.11 (mean ± standard deviation), and a significant difference of 0.05 was assumed between the two groups. The estimated sample size was 76 patients per group with a power of 80% and type-1 error of 0.05, to detect the superiority of HFNO to FMV in terms of the highest ORI. However, because this was the first randomised controlled trial to monitor the ORI throughout the anaesthesia induction period and because the ORI is a new parameter and the risks of data collection failure, inter-individual variability, and patient dropout was high, the sample size was increased to 100 patients per group.

### Statistical analysis

Continuous variables are reported as mean ± standard deviation, and categorical variables are reported as numbers (percentages). Continuous variables were analysed using Student’s t-test or the Mann–Whitney U test, as appropriate. Categorical variables were analysed using the chi-square test or Fisher’s exact test. ORI and SpO_2_ during the induction process were analysed using Student’s t-test. All analyses were performed using R package version 4.1.0 statistical software (http://www.R-project.org; The R Foundation for Statistical Computing, Vienna, Austria). Statistical significance was defined as a two-sided P-value < 0.05.

## Results

**Fig. 1:**
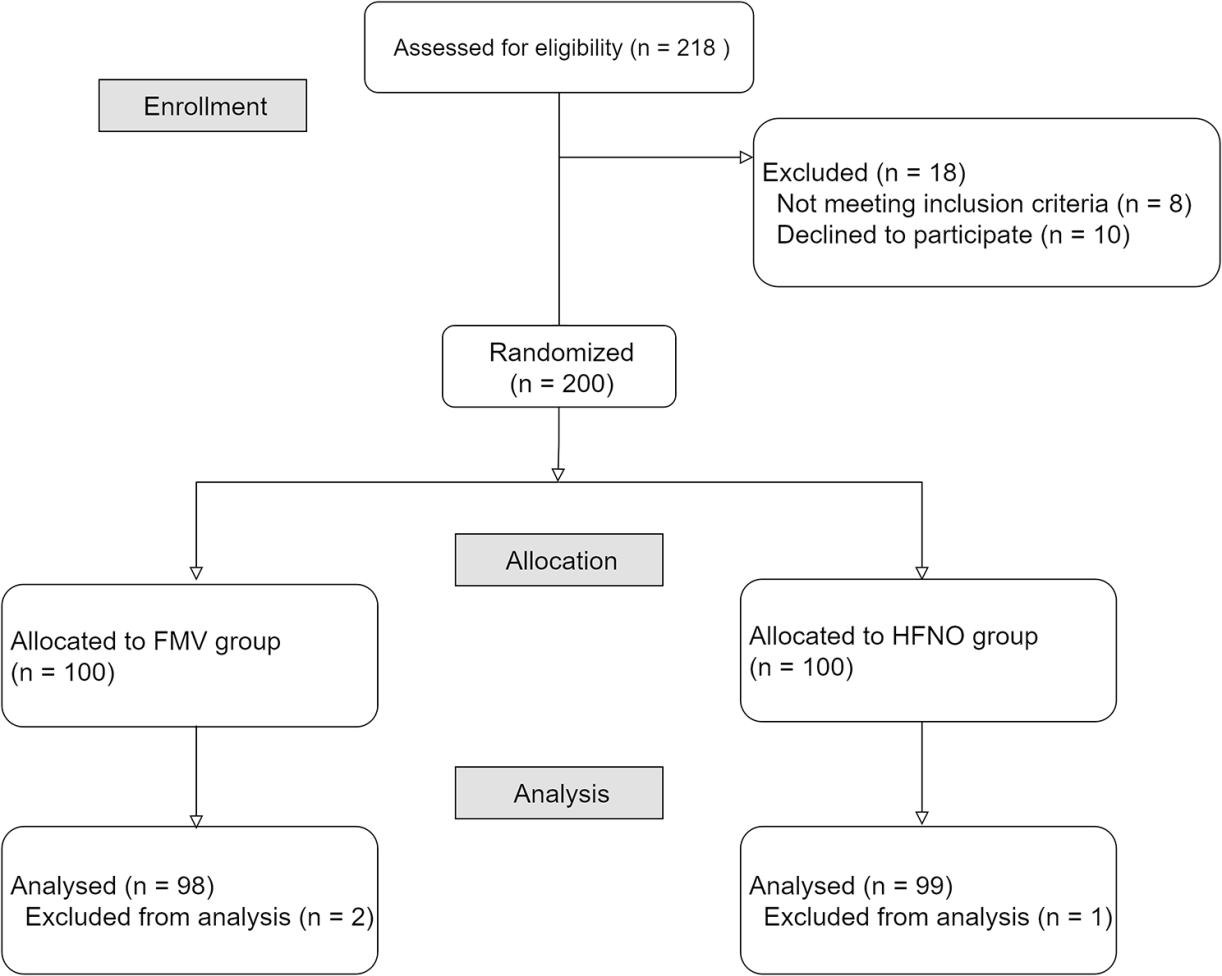
Patient enrolment flowchart Among 218 patients scheduled for elective surgery under general anaesthesia who required orotracheal intubation, eight did not meet the inclusion criteria and 10 declined to participate. Three patients were excluded from the study due to technical problems regarding the recording of vital signs, including the oxygen reserve index. The final analysis included 197 patients

A total of 197 patients were included in the final analysis (Fig. 1). Patients in the HFNO group had a greater mean height than those in the facemask oxygenation group (P = 0.027), and there were significantly more males in the HFNO group (P = 0.017, Table 1). Except for three patients, all those enrolled in this study were scheduled to undergo otorhinolaryngologic surgery. In addition, two of the enrolled patients were scheduled for surgical dental extraction and one for left lower lobectomy of lung. Overall, 3.0% of the patients were diagnosed with coronary artery disease and 1.0% with chronic obstructive pulmonary disease. One patient was diagnosed with obstructive sleep apnea by a physician.

**Table 1:** Characteristics of the patients in the two groups

		FMV (n = 98)	HFNO (n = 99)	P-value
Age (years)		51.47 ± 15.6	48.74 ± 17.4	0.248
Sex: male		49 (50.0%)	67 (67.7%)	0.017
Height (cm)		164.69 ± 9.5	167.53 ± 8.3	0.027
Weight (kg)		66.53 ± 12.3	67.45 ± 11.2	0.583
BMI (kg/m^2^)		24.38 ± 3.3	23.94 ± 3.2	0.332
American Society of Anaesthesiologists physical class	1	34 (34.7%)	49 (49.5%)	0.078
	2	48 (49.0%)	34 (34.3%)	
	3	16 (16.3%)	16 (16.2%)	
Obstructive sleep apnoea		3 (3.1%)	0 (0.0%)	0.241
Snoring		58 (59.2%)	55 (55.6%)	0.711
Modified Mallampati score	1	59 (60.2%)	60 (60.6%)	0.998
	2	31 (31.6%)	31 (31.3%)	
	3	8 (8.2%)	8 (8.1%)	
	4	0 (0.0%)	0 (0.0%)	
Interincisor distance (cm)		4.25 ± 0.6	4.13 ± 0.6	0.159
Hyomental distance (cm)		4.72 ± 0.8	4.76 ± 0.8	0.654
Head and neck movement	Normal	97 (99.0%)	98 (99.0%)	1.000
	Limited	1 (1.0%)	1 (1.0%)	
Upper lip bite test^a^ score	I	96 (98.0%)	98 (99.0%)	0.993
	II	2 (2.0%)	1 (1.0%)	
	III	0 (0.0%)	0 (0.0%)	

Data are presented as mean ± standard deviation, or the number of patients (percentage). ^a^Upper lip bite test was rated as class I if the lower incisors could bite the upper lip above the vermilion line, class II if the lower incisors could bite the upper lip below the vermilion line, and class III if the lower incisors could not bite the upper lip.

Patients in the HFNO group reported more discomfort than those in the facemask oxygenation group (P < 0.001, Table 2). The oxygen flow rate was adjusted to 6 L·min^− 1^ in one patient in the facemask oxygenation group and to 30–35 L·min^− 1^ in 23 patients in the HFNO group. All patients underwent successful preoxygenation with the adjusted gas flow rates.

**Table 2:** Level of comfort and intubation characteristics in the two groups

		FMV (n = 98)	HFNO (n = 99)	P-value
Level of comfort	Comfortable	95 (96.9%)	63 (63.6%)	< 0.001
	Acceptable	1 (1.0%)	13 (13.1%)	
	Uncomfortable	1 (1.0%)	18 (18.2%)	
	Intolerable	1 (1.0%)	5 (5.1%)	
Number of intubation attempts	1	96 (98.0%)	99 (100%)	0.360
	> 2	2 (2.0%)	0 (0.0%)	
Cormack–Lehane grade	1	95 (96.9%)	97 (98.0%)	0.840
	2	2 (2.0%)	1 (1.0%)	
	3	1 (1.0%)	1 (1.0%)	
Intubation time (min)		1.2 ± 0.99	1.2 ± 0.82	0.702

Data are presented as mean ± standard deviation or the number of patients (percentage).

**Fig. 2:**
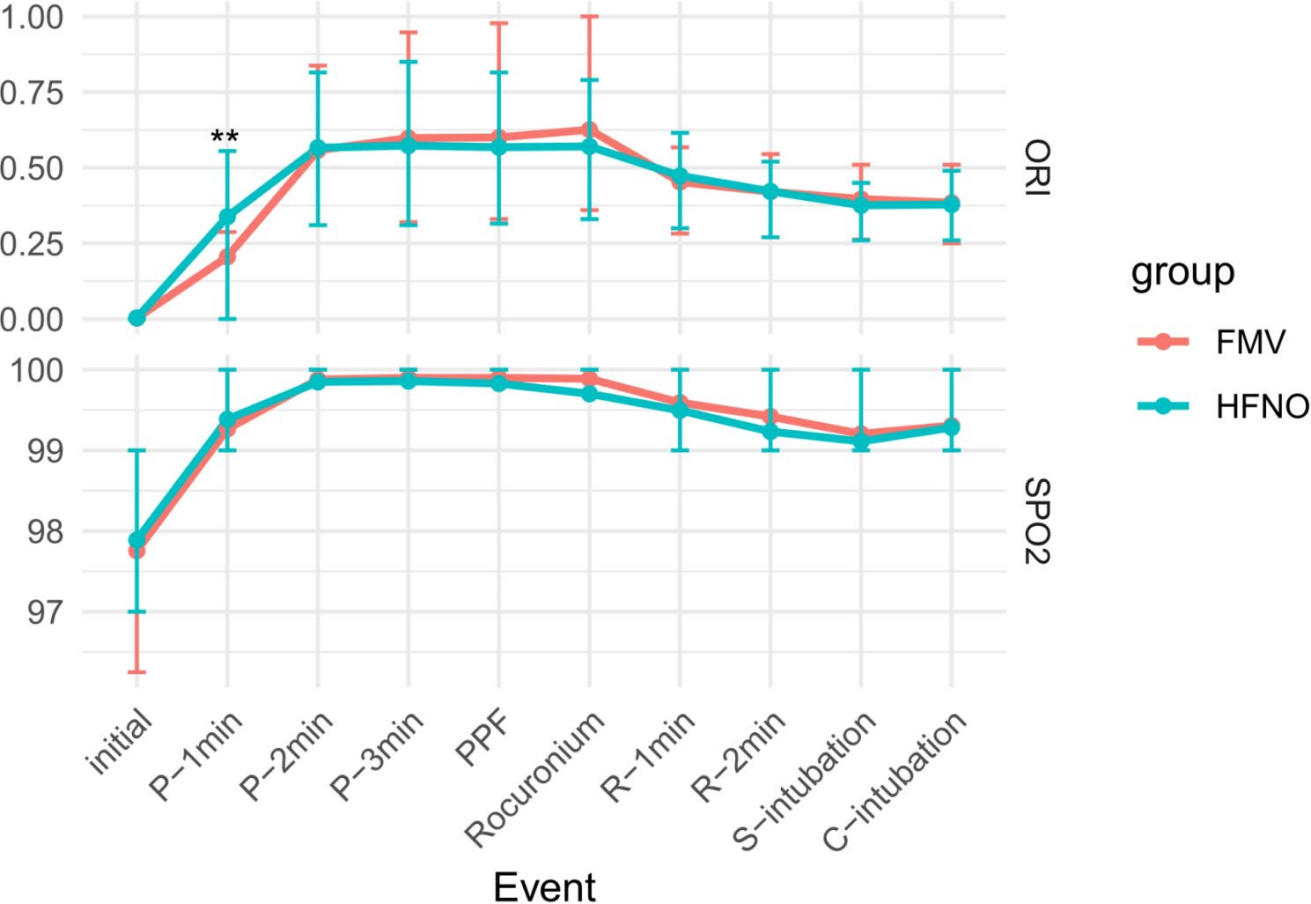
Changes in ORI and SpO_**2**_ during the anaesthetic induction period Changes in the ORI and SpO_2_ that occurred during the anaesthetic induction period in the HFNO and FMV groups are shown. ORI and SpO_2_ during the induction process were analysed using Student’s t-test. **Indicates P < 0.01 when the two groups are compared. The dots represent the mean values, and the top and bottom lines represent the standard deviations. Abbreviations: ORI, oxygen reserve index; SpO_2_, oxygen saturation; HFNO, high-flow nasal oxygenation; FMV, facemask ventilation; P-1 min, 1 min of preoxygenation; P-2 min, 2 min of preoxygenation; P-3 min, 3 min of preoxygenation; PPF, propofol injection; R-1 min, 1 min after rocuronium injection; R-2 min, 2 min after rocuronium injection; S-intubation, start of intubation trials; C-intubation, completion of intubation

**Fig. 3:**
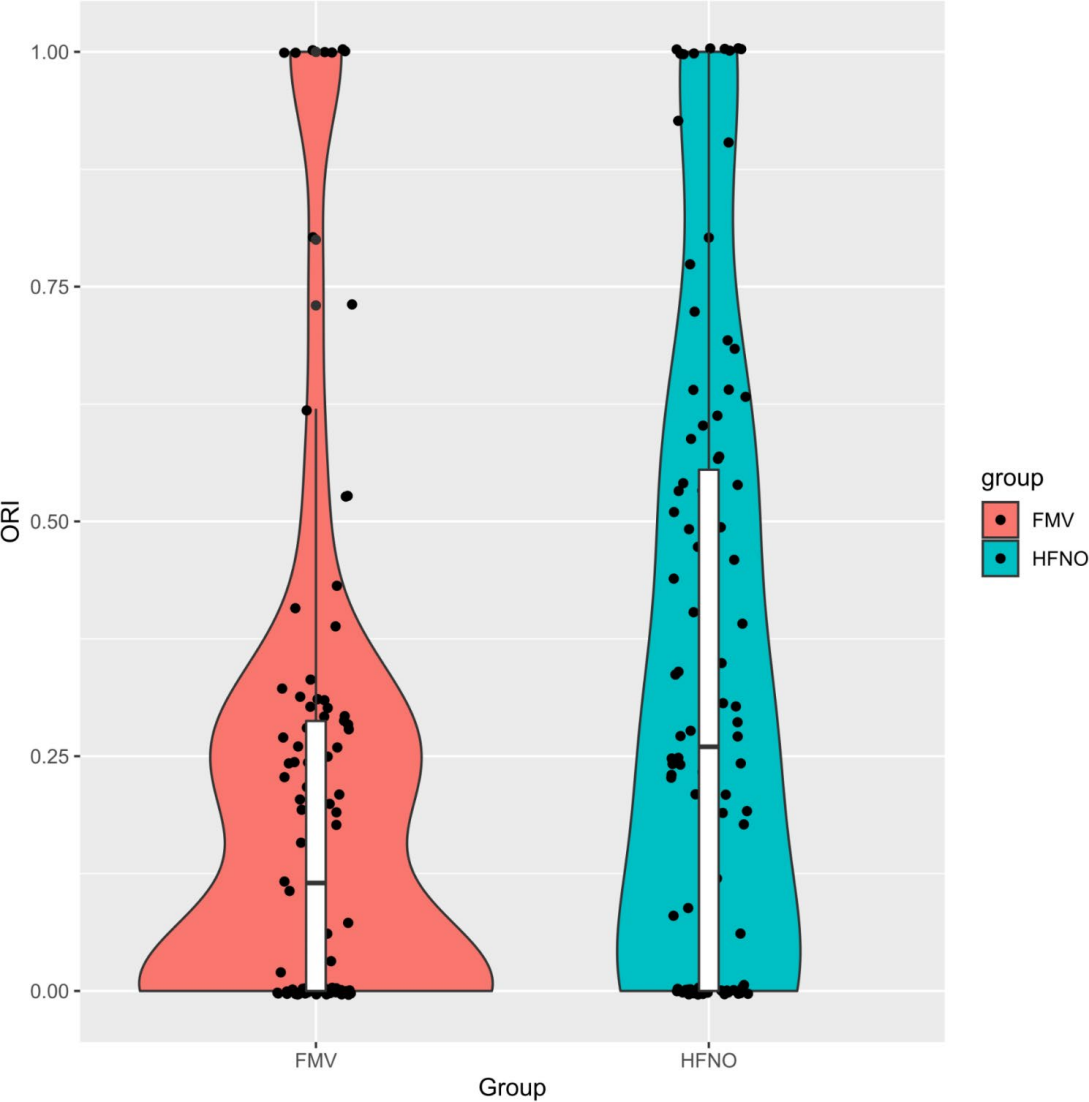
The ORI at 1 min of preoxygenation Violin plots show the distribution of the ORI at 1 min of preoxygenation for patients in the HFNO and facemask oxygenation groups. A boxplot comparing the ORI at 1 min of preoxygenation between the two groups, which was assessed using Student’s t-test (P < 0.01), is also shown. The horizontal bar represents the median, and the edges of the box represent the interquartile range, with the whiskers representing the lower quartile. Abbreviations: ORI, oxygen reserve index; HFNO, high-flow nasal oxygen; FMV, facemask ventilation

The ORI increased during preoxygenation in all patients (Fig. 2; Table 3). The ORI at 1 min of preoxygenation was significantly higher in the HFNO (0.34 ± 0.33) than in the facemask oxygenation group (0.21 ± 0.28; P = 0.003) (Fig. 3). The ORI was not significantly different between the two groups at other points during the induction of anaesthesia. At the completion of intubation, the ORI was 0 in 2.0% of patients in the HFNO group and in 6.1% of patients in the facemask oxygenation group (P = 0.136). The highest ORI was not significantly different between the HFNO (0.68 ± 0.25) and facemask oxygenation (0.70 ± 0.28; P = 0.505) groups, nor was the time required to reach the highest ORI (3.1 ± 2.2 min and 3.6 ± 2.2 min, respectively; P = 0.113).

**Table 3:** Changes in the oxygen reserve index during anaesthetic induction in the two groups

Time points	FMV (n = 98)	HFNO (n = 99)	P-value
Baseline, before preoxygenation	0.00 ± 0.00	0.00 ± 0.00	0.839
At 1 min of preoxygenation	0.21 ± 0.28	0.34 ± 0.33	0.003
At 2 min of preoxygenation	0.56 ± 0.30	0.57 ± 0.29	0.840
At 3 min of preoxygenation	0.60 ± 0.30	0.57 ± 0.29	0.553
Injection of propofol	0.60 ± 0.31	0.57 ± 0.28	0.438
Injection of rocuronium	0.63 ± 0.30	0.57 ± 0.27	0.179
At 1 min after rocuronium injection	0.45 ± 0.24	0.47 ± 0.23	0.536
At 2 min after rocuronium injection	0.42 ± 0.23	0.42 ± 0.21	0.964
Start of intubation attempt at 3 min after rocuronium injection	0.40 ± 0.21	0.38 ± 0.19	0.478
Completion of intubation	0.38 ± 0.21	0.38 ± 0.20	0.834

Data are presented as mean ± standard deviation.

The baseline mean SpO_2_ was 97.9 ± 1.4% and 97.8 ± 1.5% in the HFNO and facemask oxygenation groups, respectively (P = 0.517, Fig. 2); two patients had a baseline SpO_2_ < 96%. After 3 min of preoxygenation, the mean SpO_2_ was 99.9 ± 0.4% and 99.9 ± 0.3% in the HFNO and facemask oxygenation groups, respectively (P = 0.422). All patients had an SpO_2_ > 98% after preoxygenation.

All patients underwent successful tracheal intubation. Two patients required more than one intubation attempt, including one patient with a loose tooth and another patient with a grade III airway who required a video laryngoscope (AceScope, Acemedical Co., Korea followed by Glidescope®, Saturn Biomedical System Inc., Burnaby, BC, Canada).

## Discussion

The present study revealed that the ORI was not significantly different between patients who received HFNO and facemask oxygenation. The ORI reflects a wider range of PaO_2_ values than the SpO_2_ [10]. Szmuk et al. [13] reported that the ORI decreased as the SpO_2_ remained at 100% in 25 paediatric patients who experienced apnoea. When rapid sequence induction was performed for surgery in 16 adult patients, facemask preoxygenation increased the median ORI from 0 to 0.50 [12]. Similarly, in this study, the ORI varied during anaesthesia induction. During the 3 min of preoxygenation, the ORI increased from 0 at baseline to 0.02–1.0.

There was no significant difference in the maximum ORI obtained by the different preoxygenation methods used or in the time required to reach the maximum ORI. Pillai et al. reported that HFNO at 60 L·min^− 1^ did not result in significantly different end-tidal O_2_ than that achieved with facemask oxygenation after 3 min, which is consistent with the present study results [14]. However, Hanouz et al. reported that high-flow nasal preoxygenation at 60 L·min^− 1^ resulted in significantly lower end-tidal O_2_ than facemask preoxygenation in 50 volunteers [15]. The conflicting results may be due to the fact that the previous study included healthy volunteers and measured the end-tidal O_2_ once immediately after preoxygenation. The current study included patients who required anaesthesia, and the ORI at 1 min was significantly higher in the HFNO group, suggesting that this method induced hyperoxia more rapidly. That said, this difference was offset over time. More research using the ORI is necessary to determine whether the oxygenation reserve status differs significantly when different preoxygenation methods are used.

Herein, 23% of patients who underwent HFNO reported discomfort at a flow rate of 40 L·min^− 1^. This is consistent with the findings of a previous study wherein 40% of patients reported discomfort when 60 L·min^− 1^ of HFNO was administered [14]. In another study, 21% of patients experienced discomfort at 50 L·min^− 1^ high-flow nasal preoxygenation [8]. The flow rate used in this study was lower than that used in previous studies; nevertheless, discomfort was still reported. However, per another previous study, patients found HFNO more comfortable than facemask oxygenation [9], possibly because an oxygen flow rate as low as 30 L·min^− 1^ was used. In this study, no patients reported discomfort when the flow rate was reduced to 30 L·min^− 1^, and an appropriate preoxygenation effect was obtained at this flow rate. Therefore, it is crucial to verify that the equipment is comfortable for the patient and to adjust the flow rate appropriately during HFNO.

Endotracheal intubation was conducted 3 min after the neuromuscular block was administered, during which time the ORI decreased. The ORI was not significantly different between the two groups during this period. While mask ventilation was possible after neuromuscular blockade in the facemask oxygenation group, the ORI continued to decrease, possibly due to the ventilation/perfusion mismatch caused by atelectasis after anaesthesia induction and the decreased functional residual capacity due to muscle paralysis [16]. Further, facemask oxygenation is limited by the fact that oxygen cannot be supplied during endotracheal intubation. However, the endotracheal intubation time was as short as 1 min in this study; therefore, the disadvantages of facemask oxygenation are unlikely to have affected the patients of this study.

Although oxygen was supplied at a constant flow through the nasal cannula without ventilation, patients in the HFNO group had a similar oxygenation status during mechanical positive pressure ventilation as the facemask oxygenation group. These findings are consistent with previous results—HFNO did not cause significant differences in patient lung volume relative to positive-pressure ventilation, as measured via electrical impedance tomography [17]. Additionally, the effect of apnoeic oxygenation, in which oxygen moves from the pharynx to the alveoli and from the alveoli to the bloodstream according to the difference in blood solubility of oxygen and carbon dioxide without diaphragm movement or lung expansion, is supported by these results [18]. If ventilation is not possible, HFNO is an alternative for providing oxygen into the pharyngeal cavity. In prior studies, HFNO was deemed useful for reducing the risk of hypoxemia when performing rapid sequence induction in pregnant women, patients undergoing emergency surgery, and those in the intensive care unit [7, 19, 20]. In this study, the ORI was similar between the two groups, suggesting that high-flow nasal preoxygenation is sufficient for approximately five minutes of apnoea after neuromuscular block injection until endotracheal intubation is completed. HFNO can maintain the oxygenation status even when the endotracheal intubation time is prolonged as it can supply oxygen continuously without interfering with the intubation attempts [3]. In the absence of tidal ventilation, increasing the pharyngeal oxygen fraction improves reoxygenation during the intubation of difficult airways [21]. The benefits of HFNO are shown by measuring the safe apnoea time [22, 23], though this is beyond the scope of the current study.

Other advantages of HFNO include facilitation of a high level of humidification in the airway and reduction of atelectasis, which is positive end-expiratory pressure effect with the mouth closed [24]. While these two outcomes were not investigated in our study, ample research has supported the favorable effect of HFNO on these outcomes [25, 26]. Despite these advantages, the application of HFNO may be limited by concerns about performing aerosol-generating procedures such as HFNO during the coronavirus disease 2019 pandemic. Hamada et al. [27] revealed that HFNO might not generate aerosols but may disperse them. Thus, it is unlikely that HFNO increases the transmission of infectious disease.

This study had some limitations. First, the ORI may differ at different flow rates of HFNO. A flow rate of 40 L·min^− 1^ was used when the patient was awake in this study, and it was increased to 70 L·min^− 1^ when the patient lost consciousness. However, the gold standard for the method of HFNO has not yet been determined [3]. Second, critically ill patients or rapid sequence induction situations were not included in this study. Critical patients often require emergency endotracheal intubation due to cardiac arrest or severe hypoxemia and have a high risk of hypoxemia. Third, after patients lost consciousness, the two-handed jaw thrust technique was used regardless of the group, and peak end-expiratory pressure was not used in the facemask oxygenation group. This reflects the standard treatment in our department for FMV. The two-handed jaw thrust movement improves upper airway patency more than the one-handed mask ventilation technique [28]. Differences in tools, manpower, and manoeuvres used in the anaesthesia induction process may affect the results. Moreover, even after following appropriate randomization protocols, demographic factors such as sex and height were significantly different in the two groups. Finally, the respiratory rate during preoxygenation was not investigated in this study. However, the flow rate in both groups was high enough to offset the decrease in flow rate associated with a possible decrease in respiratory rate during spontaneous breathing.

## Conclusions

HFNO during the general anaesthesia induction process required for endotracheal intubation improves and maintains the ORI similarly to the facemask oxygenation method. Therefore, HFNO can be used to secure oxygenation reserve during anaesthetic induction.

## Data Availability

The datasets analysed during the current study are available from the corresponding author on reasonable request.

## Abbreviations

FMV: Facemask ventilation
HFNO: High-flow nasal oxygenation
ORI: Oxygen reserve index
THRIVE: Transnasal humidified rapid-insufflation ventilatory exchange
